# Abscisic acid is a substrate of the ABC transporter encoded by the durable wheat disease resistance gene *Lr34*

**DOI:** 10.1111/nph.15815

**Published:** 2019-04-22

**Authors:** Simon G. Krattinger, Joohyun Kang, Stephanie Bräunlich, Rainer Boni, Harsh Chauhan, Liselotte L. Selter, Mark D. Robinson, Marc W. Schmid, Elena Wiederhold, Goetz Hensel, Jochen Kumlehn, Justine Sucher, Enrico Martinoia, Beat Keller

**Affiliations:** ^1^ Department of Plant and Microbial Biology University of Zurich Zurich Switzerland; ^2^ Biological and Environmental Science & Engineering Division King Abdullah University of Science and Technology Thuwal Saudi Arabia; ^3^ Institute of Molecular Life Sciences University of Zurich Zurich Switzerland; ^4^ SIB Swiss Institute of Bioinformatics University of Zurich Zurich Switzerland; ^5^ Plant Reproductive Biology Leibniz Institute of Plant Genetics and Crop Plant Research (IPK) Gatersleben Seeland/OT, Gatersleben Germany

**Keywords:** abscisic acid (ABA), cereal crops, durable disease resistance, fungal pathogen, LR34 ABC transporter

## Abstract

- The wheat *Lr34res* allele, coding for an ATP‐binding cassette transporter, confers durable resistance against multiple fungal pathogens. The *Lr34sus* allele, differing from *Lr34res* by two critical nucleotide polymorphisms, is found in susceptible wheat cultivars. *Lr34res* is functionally transferrable as a transgene into all major cereals, including rice, barley, maize, and sorghum.
- Here, we used transcriptomics, physiology, genetics, and *in vitro* and *in vivo* transport assays to study the molecular function of *Lr34*.
- We report that *Lr34res* results in a constitutive induction of transcripts reminiscent of an abscisic acid (ABA)‐regulated response in transgenic rice. *Lr34‐*expressing rice was altered in biological processes that are controlled by this phytohormone, including dehydration tolerance, transpiration and seedling growth. *In planta* seedling and *in vitro* yeast accumulation assays revealed that both LR34res and LR34sus act as ABA transporters. However, whereas the LR34res protein was detected *in planta* the LR34sus version was not, suggesting a post‐transcriptional regulatory mechanism.
- Our results identify ABA as a substrate of the LR34 ABC transporter. We conclude that LR34res‐mediated ABA redistribution has a major effect on the transcriptional response and physiology of *Lr34res*‐expressing plants and that ABA is a candidate molecule that contributes to *Lr34res*‐mediated disease resistance.

## Introduction

Achieving durable field resistance against fungal pathogens is a priority of most cereal breeding programs. On average, > 10% of the global crop production is lost to diseases and pests (Chakraborty & Newton, 2011). For example, it has been estimated that 5.47 million tons of wheat (*Triticum aestivum*), worth US$979 million, are lost annually to the wheat stripe rust disease alone (Beddow *et al*., 2015). In the wheat gene pool, three genes were identified that confer durable adult plant resistance against multiple fungal diseases (Ellis *et al*., 2014). These genes were named *Lr34* (= *Yr18/Sr57/Pm38*), *Lr46* (= *Yr29/Sr58/Pm39*) and *Lr67* (= *Yr46/Sr55/Pm46*). Their expression results in partial resistance against all races of the fungal wheat pathogens causing leaf rust (*Puccinia triticina*), stripe rust (*Puccinia striiformis* f.sp. *tritici*), stem rust (*Puccinia graminis* f.sp. *tritici*) and powdery mildew (*Blumeria graminis* f.sp. *tritici*). Also, these genes cause leaf tip necrosis (LTN), a senescence‐like process that mainly develops in flag leaves of adult wheat plants (Singh, 1992; Krattinger *et al*., 2009). The partial resistance does not involve a hypersensitive response or callose deposition but is characterized by a reduced fungal growth rate, a phenotype that is also referred to as ‘slow‐rusting’ or ‘slow‐mildewing’ (Rubiales & Niks, 1995; Risk *et al*., 2012). Hence, the resistance conferred by *Lr34*,* Lr46*, and *Lr67* is different from most other disease resistance mechanisms that often result in complete but race‐specific resistance linked to hypersensitive response (Dodds & Rathjen, 2010). The *Lr34* gene has been used in wheat breeding for more than a century, and no pathogen adaptation has been recorded so far. Because of its durability and broad‐spectrum specificity, *Lr34* became one of the most frequently used disease resistance genes in wheat breeding. The *Lr34* resistance and LTN are conferred by a single gene encoding a full‐size ATP‐binding cassette (ABC) transporter (Krattinger *et al*., 2009; Risk *et al*., 2012). All resistant wheat cultivars carry the same *Lr34* allele (*Lr34res*) that evolved from an ancestral, susceptible allele (*Lr34sus*) after wheat domestication by two gain‐of‐function mutations (Krattinger *et al*., 2013). *Lr34res* is functionally transferrable into all major cereals as a transgene, including barley (*Hordeum vulgare*), rice (*Oryza sativa*), maize (*Zea mays*), and sorghum (*Sorghum bicolor*) (Risk *et al*., 2013; Krattinger *et al*., 2016; Sucher *et al*., 2016; Schnippenkoetter *et al*., 2017; Boni *et al*., 2018). In these cereal species, *Lr34res* resulted in enhanced resistance against various biotrophic or hemi‐biotrophic fungal pathogens, as well as in the development of LTN. In contrast to wheat, where the *Lr34res*‐mediated phenotype only develops in adult plants, disease resistance and LTN were already visible at seedling stage in some transgenic lines. In barley, for example, *Lr34res* conferred partial resistance against barley leaf rust (*Puccinia hordei*) and barley powdery mildew (*B. graminis* f.sp. *hordei*) (Risk *et al*., 2013; Boni *et al*., 2018), whereas in rice the expression of *Lr34res* resulted in resistance against the fungal rice blast pathogen (*Magnaporthe oryzae*) in seedlings and adult plants (Krattinger *et al*., 2016). The similarity of the *Lr34res*‐mediated phenotype across different cereal species suggests a conserved molecular mechanism and implies that the LR34res substrate is common to all cereals.

ABC transporters comprise a large gene family in plants. For example, the full‐size ABCG transporter subfamily, to which *Lr34* belongs, has 20 members in rice. ABC transporters manage the active transport of various molecules across biological membranes, including heavy metals, lipids, glucosinolates, and phytohormones (Hwang *et al*., 2016). One of the major challenges of unraveling the molecular function of this protein family is that a single ABC transporter can have multiple, structurally unrelated substrates (Lu *et al*., 2015).

Here, we used transcriptomics analyses in rice as a starting point to elucidate the molecular function of the durable disease resistance mediated by *Lr34res*. We show that both the LR34res and LR34sus protein versions are able to transport ABA in an *in vitro* yeast assay, whereas only LR34res resulted in changed ABA fluxes *in planta*. Both protein versions were present at equal amounts in yeast, whereas only LR34res was detected *in planta*, suggesting a regulatory mechanism on the post‐transcriptional level.

## **Materials and Methods**

### Plant materials and growth conditions

Rice plants were grown in a growth cabinet at 28°C : 24°C, day : night, 75% humidity, and 12 h photoperiod with 600 μmol m^−2^ s^−1^ light or in a glasshouse. Plants were grown in soil (*c*. 1 l per pot) in a nutrient solution consisting of 0.1% Sequestrene^®^ Rapid (Syngenta, Dielsdorf, Switzerland) and 0.2% Wuxal fertilizer (Syngenta). Wheat and barley plants were grown in a standard glasshouse.

### Pathogen strains

*Magnaporthe oryzae* isolate FR13 was grown on oatmeal agar (50 g l^−1^ oat flakes, 2 g l^−1^ yeast extract, 10 g l^−1^ rice starch and 15 g l^−1^ agar) at room temperature in the dark. The fungus was transferred to white light/blue light (Philips TL‐D 15W BLB) for three additional days to enhance sporulation. Rice blast conidia were then harvested from plates by rinsing with sterile distilled water (H_2_O) and raking with a spatula. Spores were filtered through three layers of gauze and suspended to a final density of (1–2.5) × 10^5^ conidia ml^−1^. The powdery mildew (*B. graminis* f.sp. *hordei*) isolate K1 was propagated on living plants, and inoculations were performed by shaking the spores from a pot containing *c*. 25 sporulating plants.

### RNA sequencing

Two transgenic rice lines that had been previously described were used for the RNA sequencing (RNAseq; Krattinger *et al*., 2016). Both lines showed partial rice blast resistance at seedling and adult plant stages. Seedling leaves and wild‐type leaves were harvested when the plants were *c*. 1 month old and the fourth leaf was fully expanded. The youngest, fourth leaf was used for RNA extraction. For adult plants, the upper half of flag leaves was harvested *c*. 2 wk after anthesis. Flag leaves of lines 5 and 16 showed *c*. 0.5 cm and *c*. 2 cm of LTN, respectively. Three biological replicates were used per line and time point. Total RNA was extracted using the SV Total RNA Isolation System (Promega). Library preparation and sequencing were done at GATC Biotech, Konstanz, Germany. Between 25.8 million and 50 million 50 bp single‐end reads were produced per sample on a HiSeq 2500 sequencing system (Illumina). Reads were aligned to the *O. sativa* ‘Nipponbare’ reference genome (MSU7, rice.plantbiology.msu.edu) with tophat v.1.2 (Trapnell *et al*., 2010) allowing up to 10 alignments per read (‐g 10) and the options ‐a 8 ‐m 1 ‐i 50 ‐I 2000 ‐F 0.2. Count tables were generated with rcount (Schmid & Grossniklaus, 2015) as described previously (Schmid *et al*., 2012) but using the read length as allocation distance for calculating the weights of the reads with multiple alignments. Variation in gene expression was analyzed with a general linear model in R with the package edger (Robinson *et al*., 2010) according to a crossed factorial design with three explanatory factors GENOTYPE (transgenic or sister line), TISSUE (flag leaf or seedling leaf), and LINE (plant line number 5 or 16). Genes differentially expressed between specific conditions were identified with pairwise comparisons using tagwise dispersion estimates and Benjamini–Hochberg multiple testing corrections. Genes with an adjusted *P*‐value (false discovery rate) < 0.05 and a minimal log_2_(fold change) = 2 were considered to be differentially expressed. In total, we detected sequence reads corresponding to 18 990 rice genes. To define an ‘*Lr34res*‐responsive core gene set’ we considered genes that were differentially expressed more than four‐fold (log_2_ > 2) in at least three of the four conditions (lines 5 and 16; seedling and adult plants). In addition, we removed genes with low read numbers (< 50 reads) and genes annotated as transposons.

The meta‐analysis in Genevestigator (Hruz *et al*., 2008) was performed using the condition search tool ‘perturbations’. An *Lr34res*‐responsive core gene was classified as pathogen inducible and/or drought inducible if it was responsive to the respective stress in at least two samples with a fold change of log_2_ > 2 according to the ‘perturbations’ tool. Validation of RNAseq data through semi‐quantitative reverse transcription PCR (RT‐PCR) or quantitative RT‐PCR (RT‐qPCR) was done on leaves of the *Lr34res*‐expressing rice line 19, a line that was not used for the RNAseq experiment. RNA was extracted from 42‐d‐old plants of lines 19 and 19sib using the SV Total RNA Isolation System (Promega). Complementary DNA (cDNA) was synthesized from 1 μg of RNA using the i‐Script™ cDNA Synthesis kit (Bio‐Rad). RT‐qPCR was performed on a CFX96 Touch™ Real‐Time PCR Detection system (Bio‐Rad) with the Kapa SYBR^®^ FAST qPCR Master Mix (Kapa Biosystems). One reaction included 5 μl KAPA Master Mix, 4 μl of 1 : 20 diluted cDNA template and 500 nM primers. The sequences of primers used in this study are listed in Supporting Information Table S1. *UBC1* or the sucrose synthase‐1 (*OsRSs1*) genes were used as reference genes (Wang *et al*., 1992; Krattinger *et al*., 2016).

### Dehydration stress and leaf transpiration experiments

For adult plants of lines 8 and 8sib, one plant per pot was grown in nutrient solution for 33 d. Then, pots were removed from nutrient solution and leaf rolling was used as a visual sign of dehydration stress (Bunnag & Pongthai, 2013). Plants of line 8 did not show LTN when the dehydration experiments were conducted. Transpiration rate and stomatal conductance on leaves of whole plants were measured using an LI‐6400 portable photosynthesis system with a 6400‐11 narrow leaf chamber (Li‐Cor, Lincoln, NE, USA). Measurements were done on 4‐wk‐old plants using a flow rate of 200 μmol s^−1^, a CO_2_ concentration of 380 μmol mol^−1^, and a light intensity of 2000 μmol m^−2^ s^−1^. Transpiration measurements on detached leaves were done as described previously (Mittelheuser & Van Steveninck, 1969). Briefly, flag leaves of booting tillers were detached and the FW of each leaf was determined. *Lr34res*‐expressing rice leaves did not show LTN at the time of harvesting. Leaves were incubated with their cut ends in 5 ml Eppendorf tubes containing either 5 ml H_2_O, 5 ml of a 10 μM ABA ((±)‐ABA; Sigma Aldrich) or 10 μM jasmonic acid (JA) solution ((±)‐JA; Sigma Aldrich). Methanol (MeOH) was used to prepare the hormone stock solutions, and equal amounts of 100% MeOH were added to the H_2_O control. Tubes were weighed after 0 and 24 h and transpiration was calculated as H_2_O uptake per gram leaf FW. Values were corrected for H_2_O loss by evaporation.

### ABA germination experiments

Early seedling establishment was determined as described previously (Zhao *et al*., 2015). In brief, rice seeds were dehusked and sterilized with 70% ethanol and 1.25% sodium hypochlorite (NaOCl). Rice caryopses were then washed five times with double‐distilled H_2_O and plated on Petri dishes containing &frac12; Murashige and Skoog medium with or without 5 μM ABA. The ABA stock solution was prepared in MeOH, and equivalent amounts of 100% MeOH were added to the control plates. Plates were then placed upright at room temperature in the dark, and seedling growth was evaluated after 7 d. The optimal ABA concentration of 5 μM was determined in an initial experiment with an ABA concentration gradient.

### Tritiated‐ABA accumulation assay in rice seedlings

Rice seeds were dehusked and sterilized with 70% ethanol and 1% NaOCl. Rice caryopses were then cold‐treated in sterile H_2_O in the dark at 4°C for 24 h and subsequently shifted to continuous light at 30°C for 40–72 h until all germinated seedlings reached the same growth stage. For the accumulation experiment, germinated seedlings were incubated in 300 μl of a bathing solution (10 mM potassium chloride, 10 mM Mes, pH 6.05) containing 1.5 nM tritiated ABA (^3^H‐ABA, ART 1186; American Radiolabeled Chemicals, St Louis, MO, USA) or 1 : 5000 (v/v) diluted tritiated JA (^3^H‐JA, ART 2152; American Radiolabeled Chemicals) for 30 min at 30°C under white light. After incubation, seedlings were washed three times with 500 μl ice‐cold bathing solution and radioactivity was determined with a liquid scintillation counter.

### Heterologous expression of *Lr34* in yeast and ABA loading assays

Full‐length cDNA of *Lr34res* was amplified from the binary vector *p6U:cLr34res* (Risk *et al*., 2013) and a hemagglutinin (HA)‐tag was introduced at the 3′ end of the cDNA using primer C‐HALr34 (5′‐GGG CGG CCG CTT AAG CGT AAT CTG GAA CAT CGT ATG GGT ACC TCT TCT GGA AAT TAA G‐3′). Amplification was performed with Herculase II Fusion DNA polymerase (Agilent Technologies, Santa Clara, CA, USA). The PCR fragment was cloned into the *Not*I site of the *Escherichia  coli* yeast shuttle vector pNEV‐N (Sauer & Stolz, 1994), resulting in *pNEV:cLr34res‐HA*. For the generation of the *Lr34sus‐HA* construct, *pNEV:cLr34res‐HA* and *pGY:Lr34sus* were subsequently digested with *Nru*I and *Bst*EII to exchange the polymorphic sites between *Lr34res* and *Lr34sus*. Resulting fragments were re‐ligated using the TaKaRa Ligation Kit LONG (TaKaRa Bio Co., Otsu, Japan), The constructs were then transformed into yeast strains W303 (Ralser *et al*., 2012) and YMM12 (Alejandro *et al*., 2012) using electroporation and selected on uracil‐minus minimal media. ^3^H‐ABA loading tests were performed as described previously with minor modifications (Kang *et al*., 2015). In brief, yeast cells were precultured in minimum salt–glucose medium in the absence of uracil (SG‐URA) at pH 5.5 overnight and resuspended in fresh SG‐URA media at OD_600_ = 0.2. Cultured cells were harvested by centrifugation at the mid‐log phase (up to OD_600_ = 1.0) after 4–8 h incubation. Pellets were washed twice using cold SG‐URA medium (pH 6.3) and then resuspended in same medium at an OD_600_ of 10. ^3^H‐ABA (4.5 nM, 7.4 kBq, 1.63 TBq mmol^−1^) and 45 nM unlabeled ABA were added to the cell suspension and gently mixed. For measurements, 100 μl of cell suspension were filtered through nitrocellulose membranes and the cells remaining on the filter were washed twice with 2 ml of ice‐cold media at each time point. The radioactivity on the filter was determined using a liquid scintillation counter. For ABA accumulation assays in the presence of ABC transporter inhibitors, yeast cells were preincubated with the respective inhibitor for 20 min. Then, they were incubated for 10 min in the same medium with radiolabeled ABA as already described. For ABA accumulation assays in the presence of various unlabeled chemicals, yeast cells were incubated with 3 μM of the respective chemical for 10 min in the same medium with 50 nM radiolabeled and unlabeled ABA mixture as already described.

### Cloning and transformation of genomic *HA‐Lr34res* and *HA‐Lr34sus* alleles into barley

The N‐terminal HA‐tag was added to the genomic *Lr34res* and *Lr34sus* constructs by homologous recombination in yeast. To do so, the binary plasmid p6U (DNA Cloning Service, Hamburg, Germany) was digested with *Eco*RI (New England Biolabs, Ipswich, MA, USA) and the Leu2 cassette was amplified on the template plasmid pRS305 using primers dst236 (5′‐CTC CAC GAA AAT ATC CGA ACG CAG CAA GAT TGG GTC CTT TTC ATC ACG TGC‐3′) and dst237 (5′‐TGC CCA GGC AAG ACC GAG ATG CAC CGC GAT GCG GCC GCC ACC GCG GT‐3′). This created a yeast compatible vector, called p6Uyeast. The p6Uyeast vector was linearized using *Sfi*I, and different PCR products (Table S1) based on *p6U:*:*Lr34res* (Risk *et al*., 2013) were transformed into yeast strain RGSY for homologous recombination. The p6Uyeast plasmids with the different alleles were used for *Agrobacterium*‐mediated stable transformation of barley cv Golden Promise as described previously (Hensel *et al*., 2008, 2009).

### Protein extraction, sodium dodecyl sulfate polyacrylamide gel electrophoresis, and Western blot

Total protein extracts were obtained by grinding barley leaves in liquid nitrogen (N) using a mortar and pestle. Then, 3 ml of extraction buffer (100 mM sodium chloride, 50 mM Tris hydrochloride pH 8, 25 mM sucrose, 5 mM EDTA, 10% glycerol, 1% Triton X‐100, 5 mM dithiothreitol and one tablet of cOmplete™ protease inhibitor cocktail (Sigma‐Aldrich) per 10 ml extraction buffer) was added per 1 g of leaf material. Soluble proteins were separated from debris by two centrifugation steps at 15 000 ***g*** for 20 min and 10 min. Enrichment of the membrane fraction was achieved by ultracentrifugation (100 000 ***g***) using an Optima Xpn 100 ultracentrifuge (Beckman Coulter, Brea, CA, USA). Protein concentration measurement was done by Bradford assay using Protein Assay Dye Reagent Concentrate (Bio‐Rad) and a Spectra Max 190 spectrometer (Bucher Biotec, Basel, Switzerland). Equal protein amounts (24 μg) were separated on a 6.5% acrylamide/bis‐acrylamide gel (Bio‐Rad) and blotted to a nitrocellulose membrane (Amersham™ Protran™ 0.2 μm NC; GE Healthcare Life Sciences, Marlborough, MA, USA) using the Mini‐Protean II system (Bio‐Rad). Blots were incubated with 1 : 1000 rat monoclonal antibody (Anti‐HA‐Peroxidase High Affinity; Roche). Signals were detected using the WesternBright™ Quantum kit (Advansta, San Jose, CA, USA) and quantified with a Fusion FX6‐XT‐820.EPI camera and the evolutioncap software (Vilber Lourmat/Witec AG, Sursee, Switzerland).

### Analysis of ABA‐deficient crosses

The *Lr34res‐*expressing line 19 in the genetic background of ‘Nipponbare’ (Krattinger *et al*., 2016) was crossed with the *OsABA8ox1* overexpressing line 27‐3 and the corresponding wild‐type ‘Toyohikari’ (Mega *et al*., 2015).

The *M. oryzae* leaf sheath assay was performed as described previously (Saitoh *et al*., 2012; Krattinger *et al*., 2016). In brief, *c*. 3 cm long leaf sheath segments of 5‐wk‐old plants were filled with a conidial spore suspension (1 × 10^5^ spores ml^−1^) of the isolate FR13. The leaf sheaths were incubated in the dark for 28 h at room temperature in humid Petri dishes. The plant tissue was fixed and stained by boiling for 1 min in lactophenol–trypan blue (30 ml ethanol, 10 ml glycerol, 10 ml lactic acid, 10 mg trypan blue, and 10 ml distilled H_2_O). Subsequently, the samples were decolored with chloral hydrate (2.5 g of chloral hydrate in 1 ml double‐distilled H_2_O) for at least 30 min. The appressorial sites were analyzed with a Zeiss Axio Imager Z1 microscope (Carl Zeiss AG, Feldbach, Switzerland). Each invasive hypha was assigned to one of the four levels of invasive growth according to Saitoh *et al*. (2012): level 1, invasive hypha is shorter than 10 μm without branches; level 2, 10–20 μm long invasive hypha with zero to two branches; level 3, invasive hypha is longer than 20 μm and/or has more than two branches per cell; level 4, more than one cell is infected by the invasive hypha.

### ABA concentration measurement

ABA concentrations of whole leaves were measured using the Phytodetek^®^ ABA test kit (Agdia‐Biofords, Grigny, France) according to the manufacturer's instruction. Rice seedlings were grown for 3 wk in growth cabinets. The latest fully developed leaves were harvested and immediately frozen in liquid N. Leaves of three plants were pooled for one biological replicate. Leaves were cut into *c*. 2 cm segments and then lyophilized for 24 h. The tissue was ground in 2 ml Eppendorf tubes containing two 4 mm glass beads using a Retsch^®^ M300 TissueLyser (Retsch GmbH, Haan, Germany) at 30 Hz for several minutes. ABA was extracted by adding 500 μl cold 80% MeOH to each sample and samples were mixed on an overhead shaker in the dark overnight. The extraction was repeated with 1 ml cold 80% MeOH. After the second extraction, samples were washed twice with 500 μl of cold 80% MeOH. Supernatants of the four extractions were pooled and reduced to *c*. 500 μl in a SpeedVac (Thermo Fisher Scientific, Waltham, MA, USA). Tris‐buffered saline (TBS, 1×) was then added up to 1 ml and samples were 1 : 10 diluted in 1× TBS for analysis.

## Results

### *Lr34res* induces transcripts reminiscent of an ABA‐regulated stress response in rice

**Figure 1:**
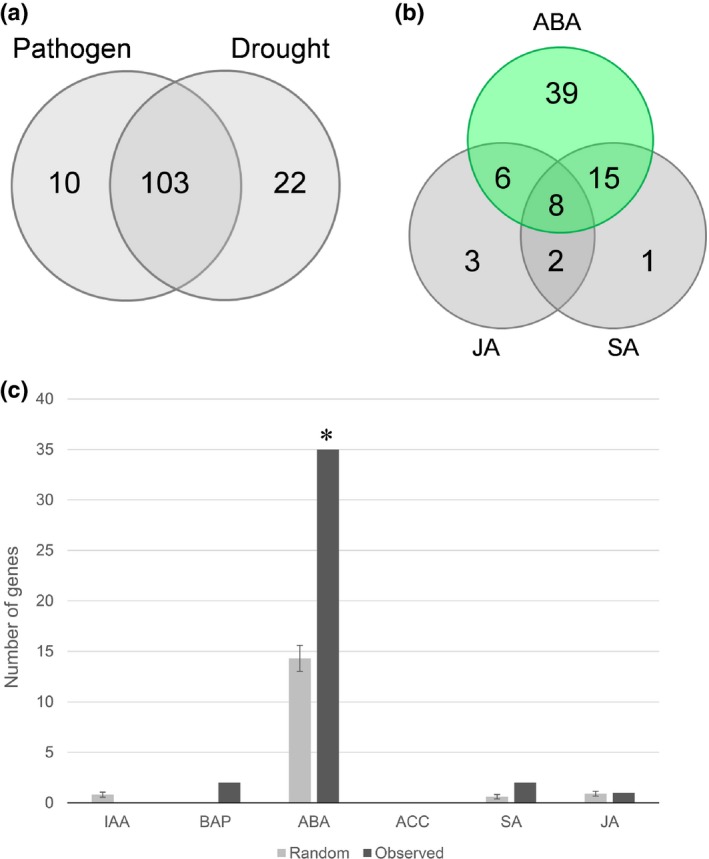
*Lr34res* constitutively induces transcripts reminiscent of an abscisic acid (ABA)‐regulated multiple stress response in rice. (a) Number of genes of the ‘*Lr34res*‐responsive core gene set’ that were responsive to pathogens and/or drought according to the gene expression database Genevestigator (Hruz *et al*., 2008). (b) Number of genes of the ‘*Lr34res‐*responsive core gene set’ that were responsive to ABA, salicylic acid (SA), and jasmonic acid (JA; Garg *et al*., 2012). (c) Comparison of gene numbers in the ‘*Lr34res*‐responsive core gene set’ (observed) to a randomly generated gene list. For the random data set, 159 rice genes (same number as ‘*Lr34res‐*responsive core gene set’) were randomly selected from the 18 990 rice genes detected in our transcriptomics study and their hormone responsiveness determined according to Garg *et al*. (2012). * denotes a statistical significance (*P* < 0.0001) based on a chi‐square test between the observed and the mean of 10 random data sets. Genes that were responsive to multiple hormones according to Garg *et al*. (2012) were not considered. Error bars represent ± SE. ACC, 1‐aminocyclopropane‐1‐carboxylic acid; BAP, benzylaminopurine; IAA, indole‐3‐acetic acid.

Previous studies in wheat and barley revealed that *Lr34res* induces constitutive defense responses even in the absence of pathogen infection (Hulbert *et al*., 2007; Chauhan *et al*., 2015). RNAseq in transgenic rice that expressed *Lr34res* revealed an ‘*Lr34res*‐responsive core gene set’ consisting of 146 up‐ and 13 downregulated transcripts that were repeatedly detected in two independent transgenic events at seedling and adult plant stage compared with wild‐type plants (Table S2). In both transgenic events, rice blast resistance and LTN were present in seedlings and adult plants (Krattinger *et al*., 2016). Uninfected leaves were used for the transcriptomics analysis based on the previous observations that *Lr34res*‐based molecular mechanisms are constitutively activated. The rice plants used for the RNAseq experiment were grown under optimal conditions, and there were no signs of pathogen infection or abiotic stresses. Despite these stress‐free growth conditions, meta‐analyses revealed that most of the 159 differentially expressed core genes in rice were responsive to stresses (Hruz *et al*., 2008; Shaik & Ramakrishna, 2014). More precisely, many of these genes are inducible by both biotic and abiotic stresses and are therefore referred to as ‘multiple stress responsive genes’ (Figs 1a, S1). Some of the *Lr34res‐*responsive core genes have been reported to confer broad‐spectrum disease resistance or abiotic stress tolerance (Table S3). An example is *BBTI4*, a gene coding for a Bowman–Birk‐type bran trypsin inhibitor that conferred partial and broad‐spectrum resistance against the bacterial blight disease (*Xanthomonas oryzae* pv *oryzae*) when it was overexpressed in rice (Pang *et al*., 2013). *BBTI4* transcript levels were *c*. 15‐fold induced in the presence of *Lr34res* (Fig. S1c). The chitinase gene *RC24* (*OsCHIT8*) that was *c*. 90‐fold induced in *Lr34res‐*expressing rice plants (Fig. S1c) has been reported to confer field resistance to stripe rust in transgenic wheat (Huang *et al*., 2013). Other genes that were highly upregulated by *Lr34res* are known to confer tolerance to drought and salinity, such as *OsLEA3‐1*,* OsRNS4*,* OsMIOX*, or *OsRCI2‐5* (Table S3; Xiao *et al*., 2007; Duan *et al*., 2012; Li *et al*., 2014; Zheng *et al*., 2014). In wheat, it has been previously observed that the *Lr34res*‐responsive genes were associated with ABA inducibility (Hulbert *et al*., 2007). We therefore compared the rice ‘*Lr34res*‐responsive core gene set’ with two transcriptomics studies performed after hormone treatments in rice and the model grass *Brachypodium distachyon* (Garg *et al*., 2012; Kakei *et al*., 2015). Similar to wheat, the core gene set of rice was more similar to an ABA‐regulated response than to salicylic acid (SA)‐ or JA‐regulated responses (Fig. 1b,c; Tables S4, S5). In summary, transcriptomics studies in wheat, barley, and rice point to a common mechanism of *Lr34res* that involves the constitutive induction of transcripts reminiscent of an ABA‐regulated stress response.

### *Lr34res*‐expressing rice plants show alterations in several ABA‐regulated traits

We performed several physiological experiments to test for a correlation between the ‘*Lr34res*‐responsive core gene set’ and ABA‐mediated phenotypes. Krattinger *et al*. (2016) identified two basic types of *Lr34res* transgenic rice lines depending on their *Lr34res* expression patterns. One line (line 8) had low *Lr34re*s expression levels and mainly developed LTN at adult plant stage, whereas the strong *Lr34res*‐expressing lines 5, 11, 16, and 19 developed LTN already at seedling stage. Rice blast resistance was observed in all lines in seedlings and adult plants. The strong *Lr34res*‐expressing lines are phenotypically very similar. Because of their reduced seed production, which is associated with reduced tiller number and sterility caused by the strong expression of *Lr34res*, these lines were used interchangeably for the various physiological experiments. For line 8, no negative impact on plant vigor was observed (Krattinger *et al*., 2016).

**Figure 2:**
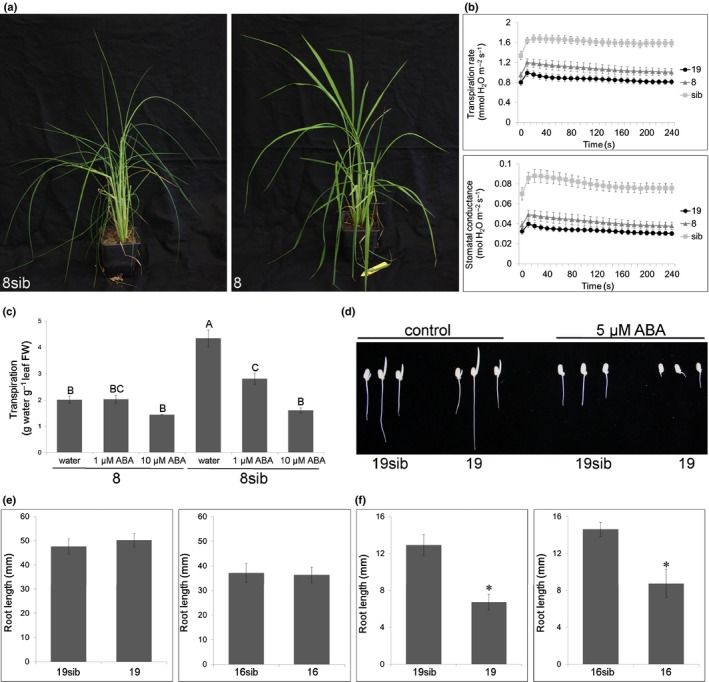
*Lr34res‐*expressing rice plants show alterations in abscisic acid (ABA)‐regulated processes. (a) Representative examples of line 8sib and the low‐expressing *Lr34res* line 8 after 4 d of dehydration stress. The leaf rolling in line 8sib is a sign of dehydration stress. (b) Transpiration rate and stomatal conductance measured on leaves of 4‐wk‐old plants. *n* = 5–15 biological replicates, mean ± SE. (c) Transpiration rate in detached flag leaves of *Lr34res* line 8 and 8sib incubated in water, 1 μM ABA, or 10 μM ABA. Letters indicate treatments with similar transpiration rates (*P* > 0.05, Tukey's honest significance test, *n* = 5 biological replicates). (d) Representative example of early seedling establishment of rice lines 19 and 19sib on &frac12; Murashige and Skoog (&frac12;MS) medium with and without ABA. The three examples per line and treatment were selected to reflect the entire range of root lengths observed. (e, f) Root length of *Lr34res*‐containing seedlings compared with their respective sibs on &frac12;MS medium with no ABA (e) and 5 μM ABA (f) after 7 d. *N* = 3, *n* = 41–64 biological replicates, mean ± SE, *, *P* < 0.01 compared with sib (Student's *t*‐test).

ABA regulates many biologically important processes, including stomatal closure and drought tolerance (Kuromori *et al*., 2014). The strong *Lr34res*‐expressing line 19 and the weak *Lr34res*‐expressing line 8 both showed increased dehydration tolerance in glasshouse conditions, which was linked to reduced transpiration rates and reduced stomatal conductance (Fig. 2a,b). The decreased transpiration rate of *Lr34res*‐expressing rice plants was phenocopied in detached wild‐type leaves by the addition of exogenous ABA (Fig. 2c). ABA is also a repressor of seed germination and early seedling growth (Zhao *et al*., 2015). Growth of *Lr34res* lines 16 and 19 on medium containing 5 μM ABA resulted in a *c*. 50% reduction of root length compared with sib lines (Fig. 2d–f), indicating hypersensitivity to exogenous ABA. In addition, LTN is associated with the upregulation of senescence‐associated genes (Krattinger *et al*., 2009; Risk *et al*., 2013), which is in line with the fact that ABA is a promoter of leaf senescence (Liang *et al*., 2014).

### *Lr34res* acts as an ABA transporter

**Figure 3:**
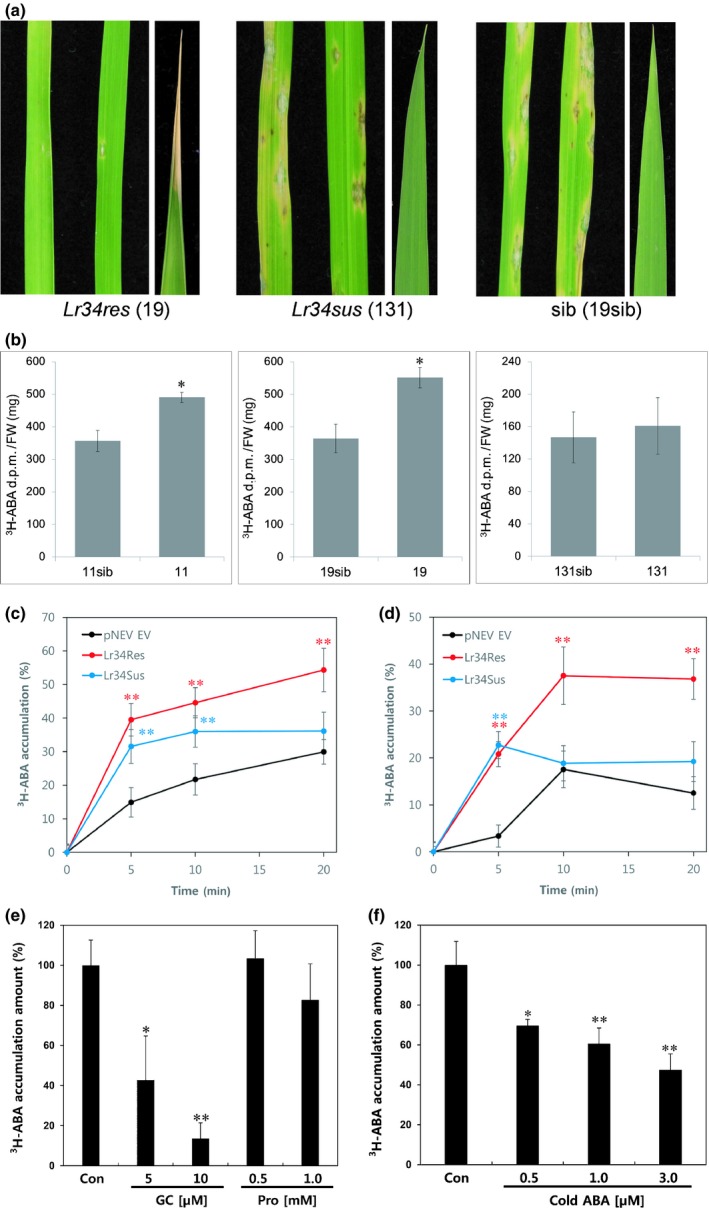
LR34 transports abscisic acid (ABA). (a) Rice blast resistance and leaf tip necrosis in 4‐wk‐old plants of *Lr34res‐*expressing line 19, *Lr34sus‐*expressing line 131 and 19sib. (b) Tritiated abscisic acid (^3^H‐ABA) accumulation (disintegrations per minute, d.p.m.) in rice seedlings of lines 11, 19, and 131 compared with respective sib lines. *N* = 3, *n* = 13–23, mean ± SEM. *, *P* < 0.01 compared with sib (Student's *t*‐test). (c) Relative ^3^H‐ABA accumulation in yeast strain W303 expressing *Lr34res* or *Lr34sus*. Mean ± SE, **, *P* < 0.01 compared with empty vector (EV) control. The graphs show averaged results of three independent experiments. (d) Relative ^3^H‐ABA accumulation in yeast strain YMM12 expressing *Lr34res* or *Lr34sus*. Mean ± SE, **, *P* < 0.01 compared with EV control. The graphs show averaged results of three independent experiments. (e) Relative ^3^H‐ABA accumulation in yeast strain YMM12 after preincubation with the ABC transporter inhibitor glibenclamide (GC) and the ABCC‐specific inhibitor probenecid (Pro). The control (Con) without inhibitor was set to 100%, mean ± SE. *, *P* < 0.05, **, *P* < 0.01. (f) Relative ^3^H‐ABA accumulation (50 nM) in yeast strain YMM12 in the absence (Con) or presence of cold ABA. The control (Con) without inhibitor was set to 100%, mean ± SEM. *, *P* < 0.05, **, *P* < 0.01.

Our analyses revealed that ABA‐regulated genes and physiological processes are altered in *Lr34res*‐expressing rice plants. We therefore tested for an effect of LR34res on ABA fluxes in rice seedlings incubated in radiolabeled ^3^H‐ABA. Both *Lr34res*‐expressing lines 11 and 19 accumulated significantly more ABA than their respective sibs after 30 min. In contrast, a homozygous rice line expressing *Lr34sus* did not show differences in ABA accumulation (Fig. 3a,b). The *Lr34sus* line 131 was chosen from several *Lr34sus*‐expressing events because it showed *Lr34* expression levels similar to the strong *Lr34res*‐expressing line 19 both in coleoptiles of young seedlings and in leaves of 4‐wk‐old plants (Fig. S2a–d). However, line 131 was susceptible to rice blast, did not develop LTN (Fig. 3a) and there was no induction of *Lr34res*‐responsive genes (Fig. S2b). Increased ABA accumulation was thus specific to *Lr34res*‐expressing rice lines, which correlates with the induction of ABA‐regulated genes, the physiological alterations, and disease resistance. *Lr34res* expression levels in coleoptiles of seedlings were *c*. 70‐fold lower than in leaves of 6‐wk‐old plants. Hence, the changes in ABA fluxes observed in *Lr34res*‐expressing rice seedlings occur at a very early growth stage and at very low *Lr34res* expression levels. As controls, nongerminated caryopses of lines 11 and 19 were incubated in ^3^H‐ABA and young seedlings in ^3^H‐JA. In both cases, we did not observe differences between *Lr34res‐*expressing lines and sibs (Fig. S3a,b).

To obtain additional evidence for LR34‐mediated ABA fluxes, we expressed the *Lr34res* and *Lr34sus* cDNAs in yeast strains W303 and YMM12. The latter carries loss‐of‐function mutations in eight ABC transporter genes. Beside the W303 wild‐type strain, YMM12 was chosen to reduce a possible effect of endogenous ABC transporters on the uptake experiments. In both yeast strains, we consistently found increased ABA uptake within 5–20 min when *Lr34res* was expressed compared with the empty vector control (Fig. 3c,d). In YMM12, a saturation was observed after 10 min, whereas relative ABA accumulation continued to increase in the W303 yeast strain up to 20 min. Surprisingly, we also observed a significantly higher ABA accumulation for *Lr34sus* at certain time points. The ABC transporter inhibitor glibenclamide but not the ABCC subfamily‐specific inhibitor probenecid blocked ABA accumulation by the ABCG transporter LR34 (Fig. 3e). Addition of increasing concentrations of cold ABA competed with the labeled ABA and resulted in a decrease of measurable ABA uptake (Fig. 3f). These experiments demonstrate that both the LR34res and LR34sus protein versions result in increased ABA uptake in yeast, whereas LR34res, but not LR34sus, changed ABA fluxes in plants. Similar to rice, the addition of SA and methyl jasmonate did not compete for ABA uptake (Fig. S3c). On the other hand, addition of the diterpene alcohol sclareol resulted in a *c*. 40% reduction of ^3^H‐ABA uptake (Fig. S3d), which might indicate competition for substrate binding. Sclareol was tested because it had been identified as a substrate of several ABCG transporters (Jasinski *et al*., 2001; van den Brule *et al*., 2002; Hwang *et al*., 2016).

**Figure 4:**
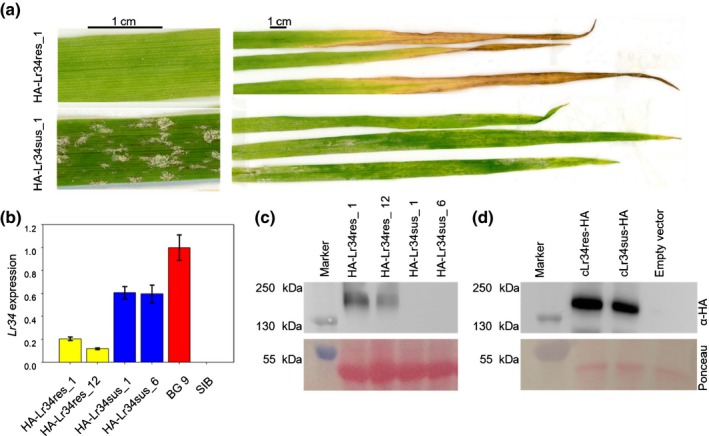
LR34res and LR34sus show differences in protein amounts *in planta*. (a) Barley leaves of the hemagglutinin (HA)‐Lr34res_1 and HA‐Lr34sus_1 events inoculated with barley powdery mildew (*Blumeria graminis* f.sp. *hordei*) 7 d post inoculation. Leaf tip necrosis developed in leaves expressing *HA‐Lr34res* but not *HA‐Lr34sus*. (b) Relative expression levels of transgenic lines expressing *HA‐LR34res* or *HA‐Lr34sus*. Expression values were normalized to *GAPDH* and set in relation to the *Lr34res*‐expressing control line BG9 (Risk *et al*., 2013). Leaves of 14‐d‐old plants were used for the quantitative reverse transcription PCR experiments. Eight T_1_ plants that were PCR positive for the respective construct were pooled. *n* = 3 technical replicates, mean ± SEM. (c, d) Western blot of extracts generated from barley leaves (c) and yeast cells (d). The PageRuler™ Plus Prestained Protein Ladder 10–250 kDa (Thermo Fisher Scientific) was used. The predicted molecular weight of LR34res is *c*. 160 kDa.

To determine the difference between LR34res and LR34sus *in planta*, we used barley plants that were transformed with *Lr34* constructs containing an N‐terminal HA tag (*HA‐Lr34*). *Lr34* expression was driven by the native wheat promoter (Risk *et al*., 2013). T_1_ plants of the two independent *Lr34res‐*expressing events HA‐Lr34res_1 and HA‐Lr34res_12 were resistant to barley powdery mildew and developed LTN, indicating that the HA tag does not interfere with the function of the LR34res protein (Fig. 4a). Similar to the *Lr34sus‐*expressing rice line 131, two HA‐Lr34sus events (HA‐Lr34sus_1 and HA‐Lr34sus_6) were susceptible to barley powdery mildew and did not develop LTN, despite having similar or even higher *Lr34* expression levels than the two HA‐Lr34res events (Fig. 4a,b). Interestingly, the HA‐LR34res protein, but not the HA‐LR34sus protein, was detectable by Western blot in extracts from barley leaves (Fig. 4c). On the other hand, both protein versions were present in yeast at similar amounts (Fig. 4d). These data indicate that both LR34 protein versions transport ABA and that the difference between LR34res and LR34sus *in planta* is regulated on the protein level through a post‐transcriptional or post‐translational mechanism. Whether the intermediate ABA uptake of LR34sus in yeast is caused by a reduced transport activity or slightly lower protein amounts could not be determined.

### Expressing *Lr34res* in an ABA‐deficient background enhances the *Lr34res* phenotype

**Figure 5:**
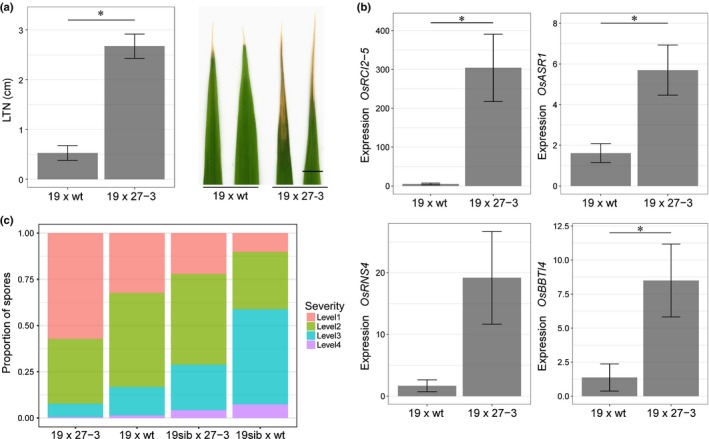
Expression of *Lr34res* in an abscisic acid (ABA)‐deficient rice background enhances the *Lr34res*‐mediated phenotype. (a) Leaf tip necrosis (LTN) on the penultimate leaf of 4‐wk‐old plants. Bar, 0.5 cm. *n* = 15–18 biological replicates; * indicates a significant difference with *P* < 0.001 (Student's *t*‐test). (b) Expression levels of the four *Lr34res*‐responsive core genes *BBTI4*, Os*RCI2‐5*, Os*ASR1*, and Os*RNS4*. Expression levels were normalized to the ubiquitin‐conjugating enzyme *UBC1*,* n* = 4 biological replicates, mean ± SE; * indicates a significant difference at *P* < 0.05 (Student's *t*‐test). (c) Classification of biotrophic, invasive *Magnaporthe oryzae* growth on rice leaf sheaths 28 h after inoculation (Saitoh *et al*., 2012; Krattinger *et al*., 2016). Levels 1–4 correspond to different lengths of invasive hyphae, with level 1 being the shortest. The *y*‐axis shows the proportion of appressorial penetration sites that belong to the different infection levels. Six biological replicates were averaged for each cross and at least 45 penetration sites were evaluated for each replicate. A statistical analysis of the disease resistance is presented in Supporting Information Fig. S5 and Table S6.

To test for a genetic link between ABA and the *Lr34res‐*mediated phenotype, *Lr34res*‐expressing rice plants of line 19 (in the genetic background of ‘Nipponbare’) were crossed to plants that overexpress the ABA catabolizing enzyme ABA 8′‐hydroxylase (*OsABA8ox1*). Plants of the *OsABA8ox1* overexpressing line E0082::OsABA8ox1 27‐3 (27‐3) had approximately four‐fold lower ABA levels and showed an ABA‐deficient phenotype compared with the respective wild‐type (‘Toyohikari’) plants (Mega *et al*., 2015). The phenotypic response of the F_1_ plants was unexpected. Although we expected a weakening of the *Lr34res*‐mediated phenotype in an ABA‐deficient background, F_1_ plants that expressed *Lr34res* and overexpressed *OsABA8ox1* (‘19 × 27‐3’) showed an enhanced *Lr34res* phenotype compared with F_1_ plants that resulted from a cross of line 19 with ‘Toyohikari’ (‘19 × Toyohikari’). This was most noticeable by longer LTN and an even stronger induction of *Lr34res*‐responsive genes (Fig. 5a,b). Also, ‘19 × 27‐3’ F_1_ plants were more resistant to *M. oryzae* than ‘19 × Toyohikari’ plants were (Fig. 5c). It has been found, however, that reduced ABA levels per se can increase resistance against *M. oryzae* in rice (Ton *et al*., 2009; De Vleesschauwer *et al*., 2013). F_1_ plants resulting from the cross ‘19sib × 27‐3’ indeed showed a certain increase in disease resistance compared with ‘19sib × Toyohikari’. The level of disease resistance in the ‘19 × 27‐3’ plants, however, was significantly higher than in the ‘19sib × 27‐3’ plants.

## Discussion

Here, we show that the phytohormone ABA is a substrate of LR34. Our *in planta* and *in vitro* uptake experiments are in agreement with the induction of ABA‐regulated genes and the ABA‐mediated physiological changes caused by the expression of *Lr34res* in rice. The results of the *OsABA8ox1* overexpressing lines further provide a genetic link between *Lr34res*‐mediated phenotypes and ABA, as LTN and *Lr34res*‐induced transcripts were strongly induced in the ABA‐deficient background. The exact molecular basis of this result needs to be determined in the future. It is likely that cell‐type or tissue‐specific differences in local ABA concentrations mediated by LR34res result in the more pronounced LTN and stronger induction of *Lr34res‐*responsive genes even when overall ABA concentrations are reduced. The increased disease resistance in the ‘19 × 27‐3’ plants could be the result of a direct or indirect action of ABA redistribution. In a direct mode of resistance induction, LR34res‐mediated ABA redistribution would cause increased disease resistance, possibly through the upregulation of the *Lr34res* induced stress response. Some of the genes induced by *Lr34res*, including *BBTI4* and *RC24*, have already been shown to confer broad‐spectrum disease resistance (Huang *et al*., 2013; Pang *et al*., 2013). It is thus possible that the sum of the *Lr34res*‐induced genes creates a more hostile environment for pathogens, resulting in the characteristic, quantitative reduction of pathogen growth rates. Interestingly, an RNAseq study focusing on pathogens revealed no transcriptional response of *P. triticina* and *B. graminis* f.sp. *hordei* to the reduction of pathogen growth caused by the presence of *Lr34res* in wheat and barley, respectively (Sucher *et al*., 2017). It is also possible that reduced local ABA concentrations in certain cells contribute to the *Lr34res*‐mediated disease resistance. On the other hand, an indirect mode of resistance activation could be the result of an additive effect of the reduced ABA levels and the transport of another substrate by LR34res. It is characteristic for ABC transporters that they can have multiple, structurally unrelated substrates (Jasinski *et al*., 2003). For example, the diterpene sclareol reduced the uptake of labeled ABA in *Lr34res*‐expressing yeast, indicating that sclareol might also be an LR34res substrate. Sclareol has been identified as an antifungal substance that serves as a putative substrate for ABC transporters (van den Brule *et al*., 2002). However, sclareol is not likely to be the physiological substrate of LR34, because it is not synthesized in monocot plants. Although the extent and time point of LTN development depends on the environment, a genetic uncoupling of *Lr34res*‐mediated LTN and disease resistance has never been observed. All the *Lr34res* loss‐of‐function mutants identified so far lost both LTN and disease resistance (Krattinger *et al*., 2009; Spielmeyer *et al*., 2013). We thus consider it likely that the *Lr34res*‐mediated disease resistance and LTN are caused by the same pathway involving an identical substrate. We therefore conclude that ABA at least partially explains the *Lr34res*‐mediated disease resistance.

The *Lr34res* allele spontaneously evolved after wheat domestication, most likely in a field of ancient farmers (Kolmer *et al*., 2008; Krattinger *et al*., 2013), and so far has not been found in wild progenitors of wheat. It is possible that the more ancient *Lr34sus* allele plays a role in ABA‐regulated stress responses and that tight regulation through post‐transcriptional or post‐translational regulation ensures its inactivation in stress‐free conditions. The spontaneous sequence changes that led to *Lr34res* might have interrupted this regulatory mechanism, which resulted in a constitutive defense response even in the absence of pathogen infection or abiotic stresses. Transgenic rice and barley plants with high *Lr34res* expression levels had a severe reduction in plant vigor and yield, most likely caused by the increased energy demand of the constitutive defense response. In wheat, it has been shown that the endogenous *Lr34res* allele can result in a slight yield penalty in high‐input farming systems with full fungicide control, whereas this allele had a beneficial effect in low‐input farming systems (Singh & HuertaEspino, 1997; Johnston *et al*., 2017). The increased energy demand could also explain why *Lr34res* was only maintained in domesticated wheat grown in agricultural ecosystems.

Several ABCG transporters in the model plant *Arabidopsis thaliana* can act as ABA transporters (Kang *et al*., 2010, 2015; Kuromori *et al*., 2010). For example, ABCG40 is an ABA importer expressed mainly in guard cells (Kang *et al*., 2010), whereas ABCG25 acts as an ABA exporter that is localized to the vascular tissue, the site of ABA production (Kuromori *et al*., 2010). These Arabidopsis ABCG transporters were identified in mutation screens for alterations in ABA‐regulated phenotypes. LR34, on the other hand, is the first example of an agronomically relevant transporter protein that is involved in ABA redistribution.

### Role of ABA in disease resistance

ABA has a multifaceted role in disease resistance and it can act as a repressor or enhancer of disease resistance depending on the pathogen, the developmental stage of the plant, and the type of affected tissue (Ton *et al*., 2009; De Vleesschauwer *et al*., 2013). For example, exogenous application of ABA increased resistance against the fungal brown spot disease of rice (De Vleesschauwer *et al*., 2010), whereas spraying of ABA on rice and barley leaves resulted in increased susceptibility against the blast pathogen *M. oryzae* (Ulferts *et al*., 2015). It has also been shown that *M. oryzae* produces and secretes ABA during the infection process to induce susceptibility, which resulted in increased ABA contents in rice leaves 24 h after infection (Cao *et al*., 2016). The ABA‐induced susceptibility to rice blast is thus linked to increased leaf ABA concentrations. In contrast, *Lr34res* did not result in an increase of whole‐leaf ABA contents (Fig. S4). The *Lr34res*‐induced disease resistance is thus likely to be caused by ABA‐redistribution in the leaf, which might result in leaf areas/tissues with increased ABA concentrations, although ABA contents might be reduced in other leaf tissues. The repressive effect of ABA on disease resistance mainly occurs through its antagonistic effect on the SA signaling pathway, which plays an important role in the defense response against pathogens. In rice, exogenous application of ABA resulted in reduced transcript levels of *OsWRKY45* and *OsNPR1*, two important co‐factors of the SA‐dependent defense response. Downregulation of these two genes was associated with increased susceptibility to pathogens (Jiang *et al*., 2010; Xu *et al*., 2013; Ueno *et al*., 2015). *OsWRKY45* and *OsNPR1*, however, were not differentially expressed in the *Lr34res* RNAseq data. This indicates that the *Lr34res‐*mediated ABA redistribution has no antagonistic effect on the SA pathway and that the *Lr34res*‐mediated disease resistance might be independent of SA‐mediated defense signaling, *OsWRKY45*, and *OsNPR1*. A recent study also reported that SA‐independent modulation of the ABA signaling pathway through the rice NAC transcription factor ONAC066 regulated disease resistance (Liu *et al*., 2018).

### A model for durable multipathogen disease resistance in cereals

Recently, the wheat multipathogen resistance gene *Lr67* was found to encode a hexose transporter (Moore *et al*., 2015). Similar to *Lr34res*, the resistant *Lr67res* allele evolved after wheat domestication as a result of spontaneous sequence changes, and only the susceptible *Lr67sus* allele was found in wild, diploid wheat progenitors. *Lr67res* and *Lr34res* both result in slow‐rusting and slow‐mildewing responses and the development of LTN. Hence, this type of durable broad‐spectrum disease resistance emerged multiple times as results of spontaneous mutations in different transporter genes. Whereas the LR67sus protein had a high affinity for glucose in yeast uptake experiments, glucose uptake was abolished by the resistant hexose transporter version. LR67res exerted a dominant negative effect on homoeologous copies of the hexose transporter. Moore *et al*. (2015) hypothesized that the dominant negative action of LR67res might increase the hexose : sucrose ratio in the leaf apoplastic space, which induces a sugar‐mediated signaling response resulting in hostile growth conditions for pathogenic fungi. Interestingly, there is a lot of crosstalk between sugar and ABA signaling (Eveland & Jackson, 2012). For example, several sugar signaling‐insensitive mutants were affected in components of ABA signaling (Arroyo *et al*., 2003). Maruyama *et al*. (2014) reported that the glyoxylate cycle, whose key enzymes isocitrate lyase and malate synthase were strongly induced by *Lr34res*, may be involved in glucose accumulation in response to drought stress in rice. A link between ABA and sugar signaling was also reported in grape (*Vitis vinifera*), where the two hexose transporters VvHT1 and VvHT5 are regulated by ABA. *VvHT1* contains multiple ABA response elements in its promoter region, and ABA plays an important role in the transcriptional regulation of *VvHT1* during infection with biotrophic fungal and oomycete pathogens (Hayes *et al*., 2010). Similarly, the expression of *VvHT5* is controlled by the ASR protein VvMSA that binds to the *VvHT5* promoter region (Cakir *et al*., 2003). Interestingly, the closest rice homologue of VvMSA is ASR1 (Perez‐Diaz *et al*., 2014), which is among the *Lr34res*‐responsive core gene set (Table S2).

Given the phenotypic similarity of *Lr34res*‐ and *Lr67res*‐mediated disease resistance, we hypothesize that the two genes trigger similar resistance mechanisms initiated either by ABA or sugar signaling, respectively. Sugars and ABA are basic molecules found in all plant species. This can explain why *Lr34res*‐mediated resistance works in different species against many fungal pathogens.

## Accession numbers

The raw RNAseq files were deposited at the Sequence Read Archive at the National Centre for Biotechnology Information with BioProject accession PRJNA317706 (SRR3348352–RR3348377).

## Author contributions

SGK, JK, EM and BK designed the research; SGK, JK, SB, RB, HC, LLS, EW and JS performed the research; GH and JK produced transgenic barley lines; SGK, JK, MDR, and MWS analyzed the data; SGK, JK, SB, RB, EM and BK wrote the manuscript. SGK, JK, SB and RB contributed equally to this work.

## Supporting information

Please note: Wiley Blackwell are not responsible for the content or functionality of any Supporting Information supplied by the authors. Any queries (other than missing material) should be directed to the *New Phytologist* Central Office.

**Fig. S1** *Lr34res* induces a multiple stress response in rice.
**Fig. S2** Characterization of the *Lr34sus* rice line 131.
**Fig. S3** LR34 changes ABA fluxes.
**Fig. S4** ABA concentrations in whole leaves of 3‐wk‐old plants of *Lr34res* containing lines 19 and 16 compared to sib lines.
**Fig. S5** Cluster analysis of the leaf sheath infection assay.
**Table S1** Primer sequences used in this study.
**Table S2** *Lr34res*‐responsive core gene set consisting of 146 up‐regulated and 13 down‐regulated genes.
**Table S3** *Lr34res*‐responsive core genes with a reported function in abiotic or biotic stress tolerance.
**Table S4** Comparison of the *‘Lr34res*‐responsive core gene set’ to a microarray study of 7‐d‐old rice seedlings incubated in solutions of 100 μM abscisic acid (ABA), 100 μM salicylic acid (SA), 100 μM jasmonic acid (JA), 50 μM benzyl aminopurine (BAP; cytokinin), 50 μM indole‐3‐acetic‐acid (IAA; auxin) or 100 μM 1‐aminocyclopropane‐1‐carboxylic acid (ACC; ethylene derivative).
**Table S5** Comparison of the ‘*Lr34res*‐responsive core gene set’ to an RNAseq analysis performed in Brachypodium seedlings incubated in 10 μM abscisic acid (ABA), 100 μM salicylic acid (SA), 30 μM methyl jasmonate (MJ), 1 μM cytokinin (CK) or 10 μM indole‐3‐acetic acid (IAA; auxin).
**Table S6** *P*‐values of the generalized linear analysis based on the leaf sheath assay.
