# Supplementary material for: Abscisic acid is a substrate of the ABC transporter encoded by the durable wheat disease resistance gene Lr34
Source: New Phytol. 2019 Apr 22;223(2):853–66. doi: 10.1111/nph.15815 (PMC6618152; doi:10.1111/nph.15815)
Supplement: Supplementary file 1 — Fig. S1 Lr34res induces a multiple stress response in rice. Fig. S2 Characterization of the Lr34sus rice line 131. Fig. S3 LR34 changes ABA fluxes. Fig. S4 ABA concentrations in whole leaves of 3‐wk‐old plants of Lr34res containing lines 19 and 16 compared to sib lines. Fig. S5 Cluster analysis of the leaf sheath infection assay. Table S1 Primer sequences used in this study. Table S2 Lr34res‐responsive core gene set consisting of 146 up‐regulated and 13 down‐regulated genes. Table S3 Lr34res‐responsive core genes with a reported function in abiotic or biotic stress tolerance. Table S4 Comparison of the ‘Lr34res‐responsive core gene set’ to a microarray study of 7‐d‐old rice seedlings incubated in solutions of 100 μM abscisic acid (ABA), 100 μM salicylic acid (SA), 100 μM jasmonic acid (JA), 50 μM benzyl aminopurine (BAP; cytokinin), 50 μM indole‐3‐acetic‐acid (IAA; auxin) or 100 μM 1‐aminocyclopropane‐1‐carboxylic acid (ACC; ethylene derivative). Table S5 Comparison of the ‘Lr34res‐responsive core gene set’ to an RNAseq analysis performed in Brachypodium seedlings incubated in 10 μM abscisic acid (ABA), 100 μM salicylic acid (SA), 30 μM methyl jasmonate (MJ), 1 μM cytokinin (CK) or 10 μM indole‐3‐acetic acid (IAA; auxin). Table S6 P‐values of the generalized linear analysis based on the leaf sheath assay. [file NPH-223-853-s001.pdf]

***New Phytologist* Supporting Information**

Abscisic acid is a substrate of the ABC transporter encoded by the durable wheat disease resistance gene *Lr34*

Simon G. Krattinger, Joohyun Kang, Stephanie Bräunlich, Rainer Boni, Harsh Chauhan, Liselotte L. Selter, Mark D. Robinson, Marc W. Schmid, Elena Wiederhold, Goetz Hensel, Jochen Kumlehn, Justine Sucher, Enrico Martinoia, Beat Keller

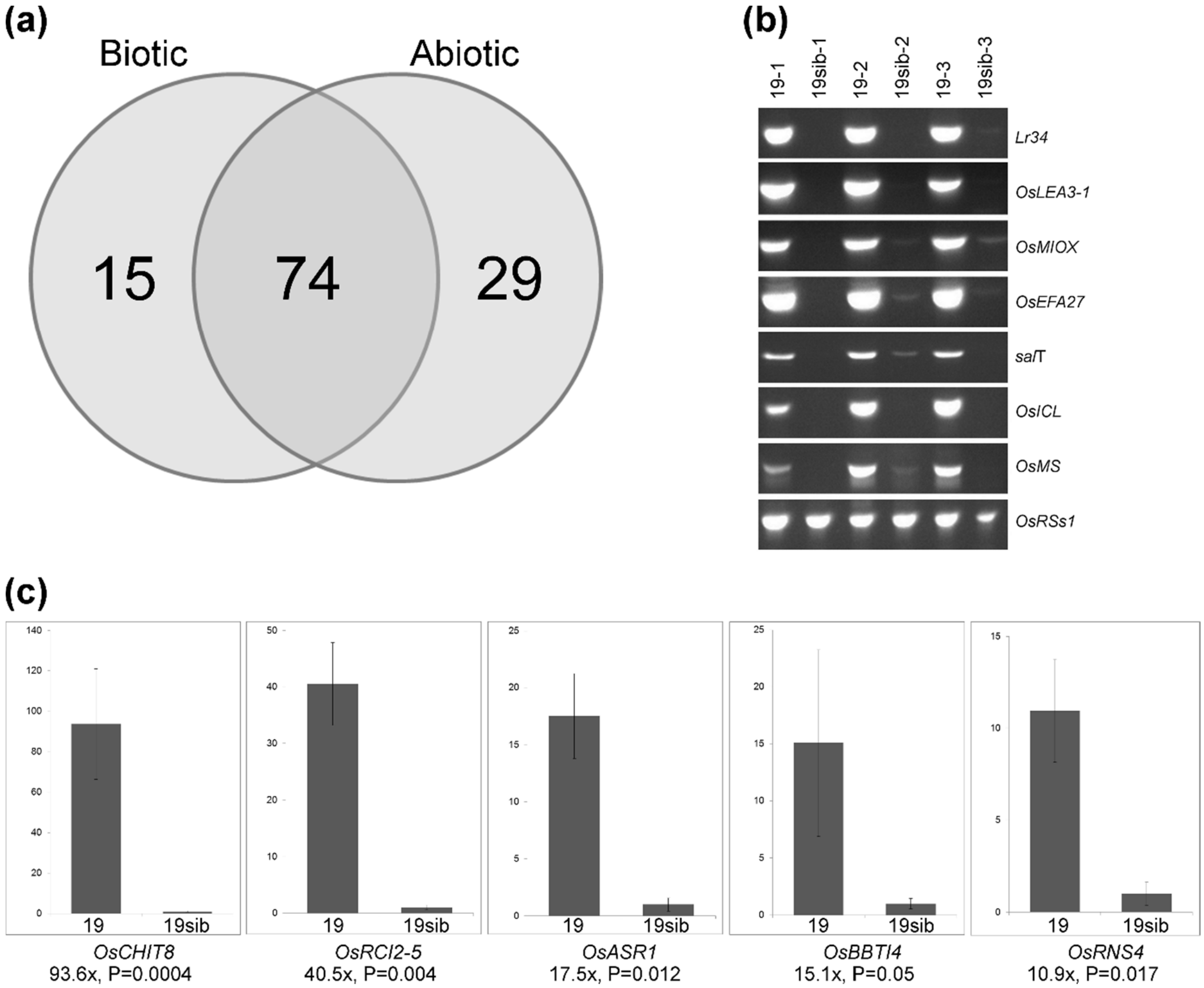

Fig. S1 *Lr34res* induces a multiple stress response in rice. **(a)** Venn diagram showing number of genes of the '*Lr34res*-responsive core gene set' that were found to be differentially expressed by biotic and/or abiotic stress in a rice meta-analysis (Shaik & Ramakrishna, 2014). **(b)** Semi quantitative RT-PCR of known stress-responsive genes. Most of these genes are not or only very weakly expressed in the sib line in a stress-free environment but they are strongly induced in the *Lr34res*-containing rice line 19. 19sib-1, 19sib-2 and 19sib-3 represent three independent biological replicates of 19sib whereas 19-1, 19-2 and 19-3 are three independent biological replicates of line 19. *OsLEA3-1* = late embryogenesis abundant 3-1; *OsMIOX* = *myo*-inositol oxygenase; *OsEFA27* = caleosin; *sal*T = salt stress-induced; *OsICL* = isocitrate lyase; *OsMS*; malate synthase; *OsRSs1* = sucrose synthase-1 (reference gene) (Wang *et al*., 1992). **(c)** Quantitative RT-PCR expression analysis of the stress-responsive genes *OsCHIT8*, *OsRCI2-5*, *OsASR1*, *OsBBTI4* and *OsRNS4*. RT-qPCRs were done on plants of the *Lr34res*-expressing rice line 19 and its sib 19sib. Relative fold changes (19sib was set to 1) and P-values (Student's t-test on $\log_{10}$ transformed expression values) are indicated. Three biological replicates were used, mean ± SEM. Details on the function of the genes used in **(b)** and **(c)** are given in Table S3.

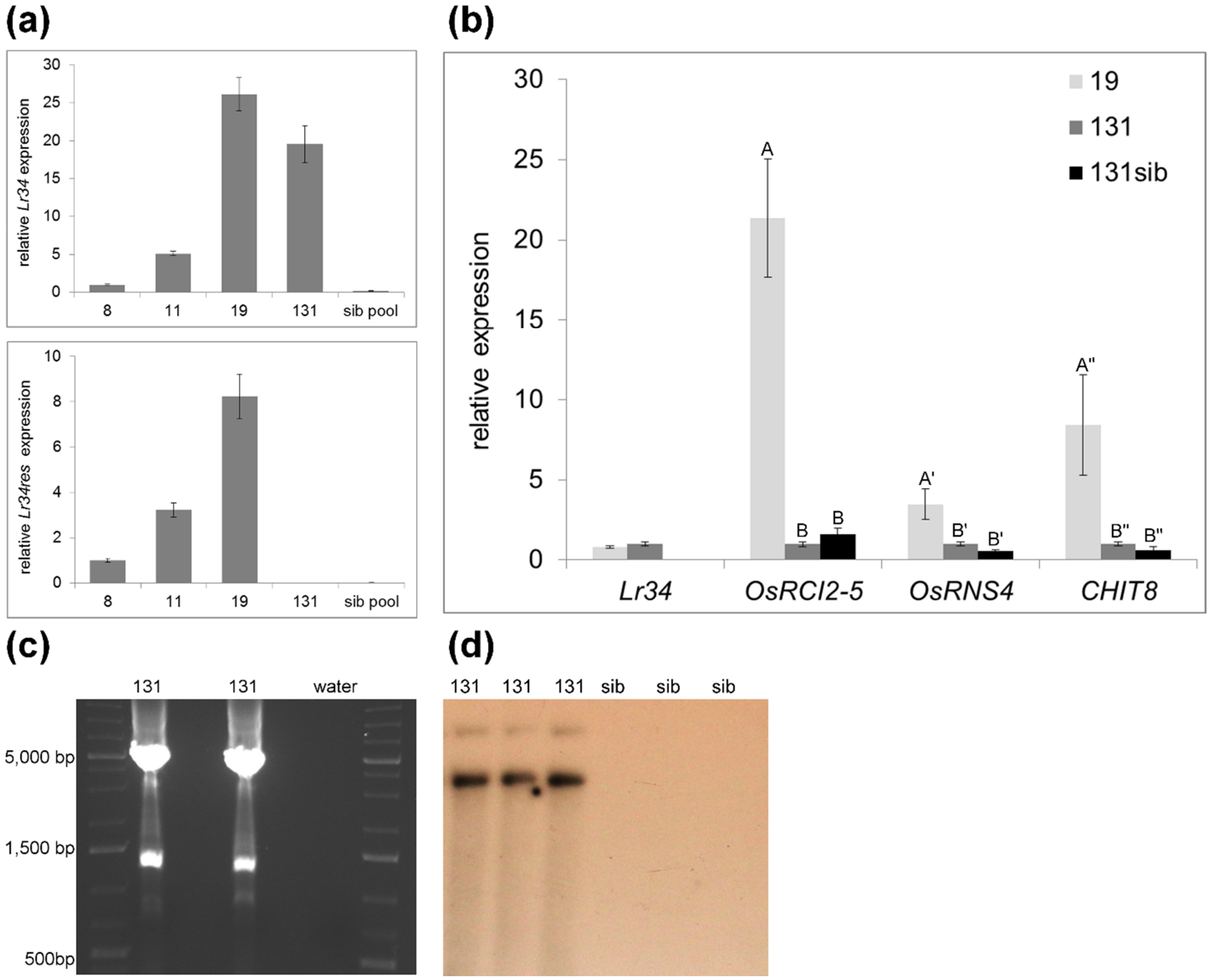

Fig. S2 Characterization of the *Lr34sus* rice line 131. **(a)** *Lr34* expression levels in coleoptiles of *Lr34res* lines 8, 11 and 19 compared to the *Lr34sus* line 131. RNA was extracted from at least 10 pooled coleoptiles. n = 3 technical replicates, mean ± SEM. A primer pair amplifying both *Lr34* alleles was used in the upper panel and a *Lr34res*-specific primer pair was used in the lower panel. **(b)** RT-qPCR for *Lr34* and the *Lr34res*-responsive genes *OsRCI2-5*, *OsRNS4* and *CHIT8* on four-week-old rice seedlings of lines 19, 131 and 131sib. n = 4 biological replicates, mean ± SEM, letters indicate lines with similar expression levels ($P > 0.05$; Tukey's honest significance test). These genes were chosen because they have a known function in stress tolerance (see Table S3) **(c)** Full-length cDNA amplification of the *Lr34sus* allele in line 131. The expected fragment size was 4,321bp. The integrity of the *Lr34sus* full-length cDNA was verified by sequencing. **(d)** Southern blot showing copy number in line 131. A probe for the selectable marker gene *HPT* was used (Risk *et al*., 2013).

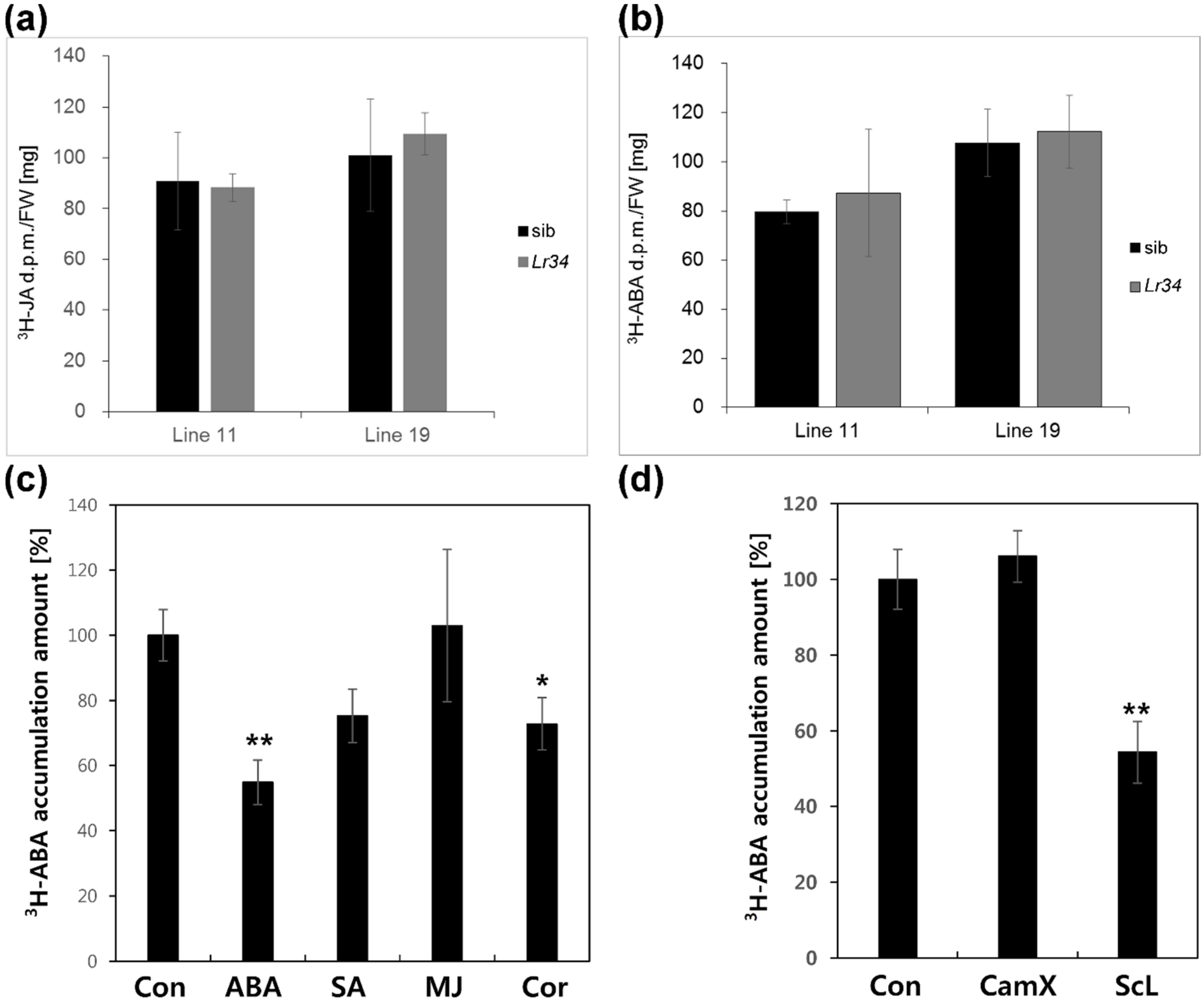

Fig. S3 LR34 changes ABA fluxes. **(a)** ^{3}H-JA accumulation (disintegrations per minute, d.p.m.) in rice seedlings of lines 11 and 19 compared to respective sib lines. N = 3, n = 6-17, mean ± SEM. **(b)** ^{3}H-ABA accumulation in non-germinated caryopses of rice lines 11 and 19 compared to sib lines. N = 3, n = 3-17, mean ± SEM. **(c)** Relative ^{3}H-ABA accumulation in yeast strain YMM12 after addition of cold phytohormones or hormone analogs [3 µM] salicylic acid (SA), metyl jasmonate (MJ), coronatine (Cor). The control (Con) without inhibitor was set to 100%, mean ± SEM. * P < 0.05, ** P < 0.01. **(D)** Relative ^{3}H-ABA accumulation in yeast strain YMM12 after addition of the indole alkaloid camalexin (CamX) and the diterpene sclareol (ScL) [3 µM]. Both components have been reported to contribute to disease resistance in Arabidopsis. The control (Con) without competitor was set to 100%, mean ± SEM. * P < 0.05, ** P < 0.01.

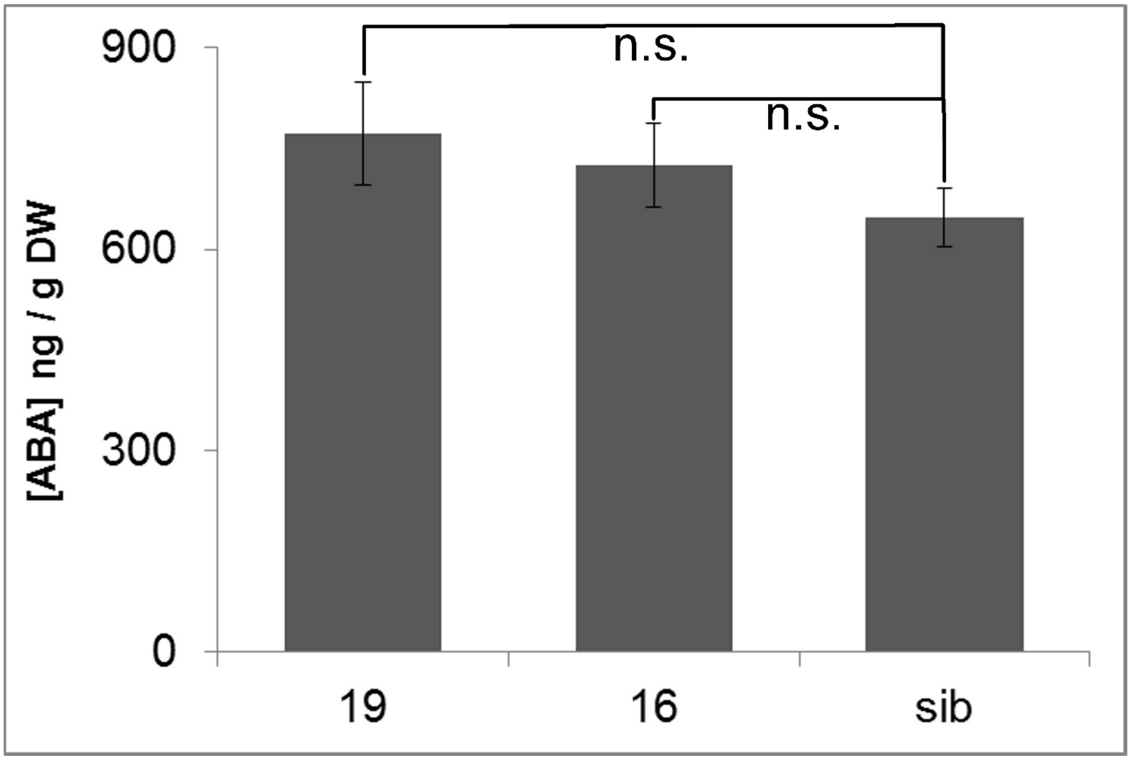

Fig. S4 ABA concentrations in whole leaves of three-week-old plants of *Lr34res*-containing lines 19 and 16 compared to sib lines. n = 3 biological replicates, mean ± SEM, n.s. = non-significant difference (Student's t-test, $P < 0.05$).

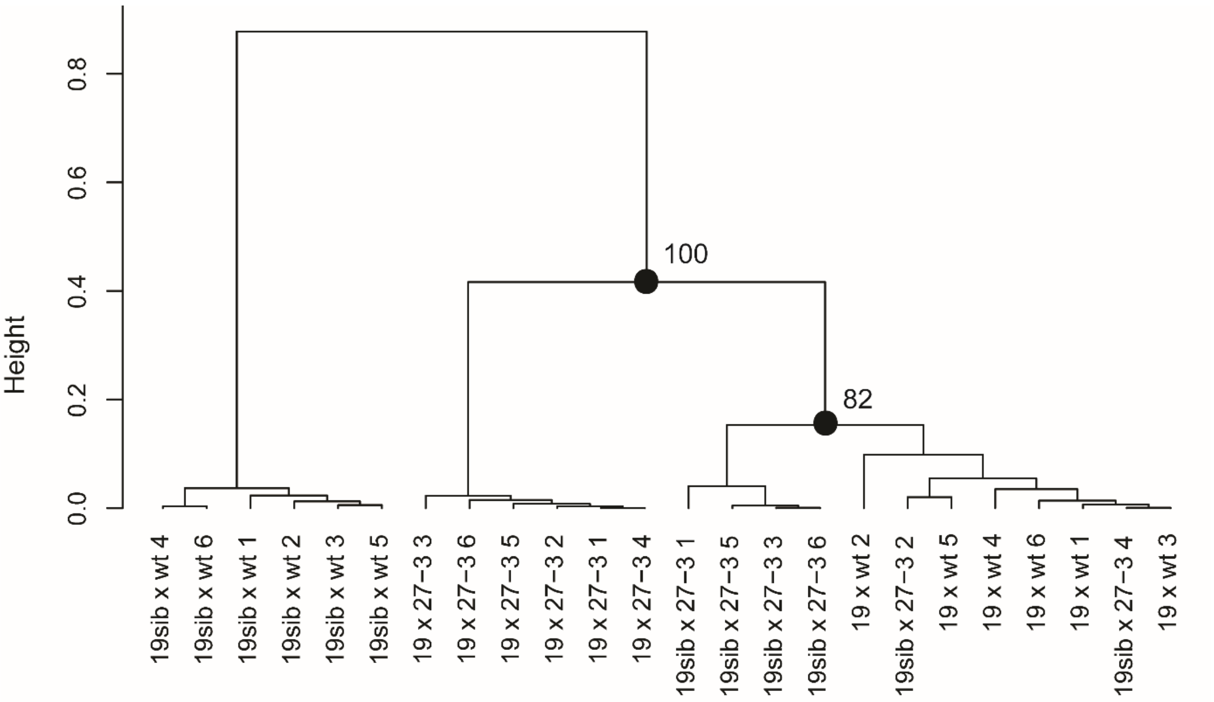

Fig. S5 Cluster analysis of the leaf sheath infection assay shown in Fig. 5c. Numbers at the edges indicate the Approximately Unbiased (AU) p-values.

Table S1. Primer sequences used in this study

semi RT-qPCR

gene name	forward primer	reverse primer	fragment size [bp]	Tm [°C]	cycle number
OsLEA3-1	GACCTCCAGCACGTCGCAGG	GTGGCAGAGGTGTCCTTGTTG	327	60	35
OsMIOX	GCCATGACGACTACATGTACC	CGATGAGTGACATGTAGTAGG	249	60	35
OsEFA27	GGTCTAAGCTACCCAACTCTG	CACGTAGAGCAATATCCACTC	282	60	30
salT	GACGCTGGTGAAGATTGGTC	CCAGTTTAATCTCTGTAGAGGTG	206	65	40
OsICL	GTGTGGCAGTTCATCACGCTC	CATGAGCCCTTGAACTGCTC	266	62	35
OsMS	GACCGCACCGTCGAGATCAC	GTGATCGTGCCGGCAACCGC	173	65	40
Lr34_full_lenght cDNA	GAGTACGGCTAGGCAATAGC	GGCAAGTAGCTATATCTGTAAC	4,321 (sus) 4,318(res)	Touch down*	35

RT-qPCR

gene name	forward primer	reverse primer	fragment size [bp]	primer efficiency	reference
Lr34sus and Lr34res rice	GACAGCGCCAGAATGGTGC	GACATCAACCCTGTCAATTC	179	105%	this study
Lr34sus and Lr34res barley	ACTGGATTCGCTTGTATGGATA	ACTGGCAGAAGAACCTTGAAACA	80	101.70%	this study
HvGAPDH	CCGGGTTCCCACTGTGGAT	TGACTAGCAACTCGGTGCGG	451	102%	this study
OsASR1	GAGCATACGCCATGCACGAG	GATCTCCTCCTTCACCCTGTG	80	105.7%	this study
OsRCI2-5	TACGGCTTGGGTATTGAGTTCTGG	CCGACGCTGGCTCTGCTC	125	95.7%	Li et al. (2014)
OsRNS4	CTCATCCTCATGTGGCCTGG	GTTGCACCTGACAACAGCTG	147	95.1%	this study
RC24/CHIT8	GTGATGACAGGGCAGTGGAC	CTCTGGTTGTAGCAGTCCAAG	209	114%	this study
BBTI4	GTGACAGCATCGTGCAGCTG	GCGGCGCACTTGTTCACCTC	277	100.80%	this study

cloning of *HA-Lr34*

	forward primer	reverse primer	fragment size [bp]	Tm [°C]	cycle number
Lr34_fragment1	CCAATATATCCTGTCAAACACTG	CAGCATAATCTGGAACATCGTATGGATACATCTCAAGATGGGTGAGTTAAATCG	2,815	55	35
Lr34_fragment2	ATGTATCCATACGATGTTCCAGATTATGCTGAGGGCCTCGCAAGAGAGAC	GGCATATGAACCCACACTCCTCAA	4,691	55	35
Lr34_fragment3	TTGAGGAGTGTGGGTTCATATGCC	TTGACTGCACGAATAACAATGGCTG	2,987	55	35
Lr34_fragment4	GCAGCCATTGTTATTCGTGCAGTC	TTGACTGCACGAATAACAATGGCTG	2,754	55	35
Lr34_fragment5	CACCATAGGCGCTTATAATGATCAGAC	ATGCAAGCTGATCCACTAG	3,314	55	35
yeast Leu casette	CTCCACGAAAATATCCGAACGCAGCAAGATTGGGTCCTTTTCATCACGTGC	TGCCCAGGCAAGACCGAGATGCACCGCGATGCGGCCGCCACCGCGGT	3,792	60	40

*Touch-down PCR (1. 98°C 3min, 2. 98°C 10sec, 3. 67°C 20sec, 72° 2min 50sec, 4. go 10x to 2. while reducing annealing temperature 1°C per cycle, 5. 95°C 10sec; 6. 55°C 20sec, 7. 72°C 2min 50sec, go 25x to 5, 8. 72° 5min)

Table S3. *Lr34res*-responsive core genes with a reported function in abiotic or biotic stress tolerance.

Locus ID	Gene name	Gene description	Log_2 FC[1)]	Reported function	Reference
LOC_Os05g46480	*OsLEA3-1*	late embryo-genesis abundant protein	11.9	Improved drought tolerance in transgenic rice	(Xiao et al., 2007)
LOC_Os06g36560	*OsMIOX*	*myo*-inositol oxygenase	3.4	Improved drought tolerance in transgenic rice	(Duan et al., 2012)
LOC_Os03g17790	*OsRCI2-5*	putative low temperature and salt responsive protein	4.1	Improved drought tolerance in transgenic rice	(Li et al., 2014)
LOC_Os09g36680	*OsRNS4*	S-like ribonuclease	3.6	Improved salinity tolerance in transgenic rice	(Zheng et al., 2014)
LOC_Os04g43200	*OsEFA27*	caleosin	8.1	Responsive to osmotic stress	(Frandsen et al., 1996)
LOC_Os07g34520	*OsICL*	isocitrate lyase	12.2	Increased expression after dehydration	(Maruyama et al., 2014)
LOC_Os04g40990	*OsMS*	malate synthase	6.5	Increased expression after dehydration	(Maruyama et al., 2014)
LOC_Os01g24710	*salT*	jacalin-like lectin domain containing protein	7.0	Responsive to drought stress	(Claes et al., 1990)
LOC_Os01g72900	*OsASR1*	ABA-, stress- and ripening-induced	2.5	Increased expression after drought stress	(Perez-Diaz et al., 2014)
LOC_Os06g51060	*RC24/CHIT8*	chitinase	4.6	Enhanced stripe rust resistance in transgenic wheat	(Huang et al., 2013)
LOC_Os01g03340	*BBTI4*	Bowman-Birk type bran trypsin inhibitor	6.1	Conferred partial, broad-spectrum resistance against bacterial blight in transgenic rice	(Pang et al., 2013)

[1)] Fold change (FC) is represented as the average of the two independent transgenic lines 5 and 16 at seedling and adult plant stages.

Table S4. Comparison of the *'Lr34res*-responsive core gene set' to a microarray study of seven-day-old rice seedlings incubated in solutions of 100 µM abscisic acid (ABA), 100 µM salicylic acid (SA), 100 µM jasmonic acid (JA), 50 µM benzyl aminopurine (BAP; cytokinin), 50 µM indole-3-acetic-acid (IAA; auxin) or 100 µM 1-aminocyclopropane-1-carboxylic acid (ACC; ethylene derivative) (Garg *et al*., 2012). In total 4,171 hormone-responsive genes were identified. 80 (79 up-regulated and 1 down-regulated) of the 159 *Lr34res*-responsive core genes were present in this list of 4,171 hormone-responsive genes. The numbers in the table list the number of genes (out of 80) that were responsive to the respective hormone. A relative quality score was assigned as following: If a gene of the 80 *Lr34re*s-responsive core genes was differentially expressed by the respective hormone in the hormone study with the same direction (up- or down-regulated) it was given the score +1. If a gene of the 80 *Lr34res*-responsive core genes was not differentially expressed in the hormone study or differentially expressed in the opposite direction it was given the score -1. The relative quality score was calculated as the sum of the individual ratings. A score of 80 indicates perfect correlation between the two data sets whereas a score of -80 indicates no correlation.

**up-regulated in *Lr34res* core gene set**	**79**	**down-regulated in *Lr34res* core gene set**	**1**	**relative quality score**	**exclusively regulated by respective hormone**	**also ABA regulated**
up-regulated by ABA	68	down-regulated by ABA	0	56	35	-
up-regulated by SA	26	down-regulated by SA	0	-28	2	23
up-regulated by JA	19	down-regulated by JA	0	-42	1	14
up-regulated by BAP	8	down-regulated by BAP	0	-64	2	6
up-regulated by IAA	4	down-regulated by IAA	0	-72	0	3
up-regulated by ACC	3	down-regulated by ACC	0	-74	0	3

Table S5. Comparison of the '*Lr34res*-responsive core gene set' to an RNAseq analysis performed in Brachypodium seedlings incubated in 10 µM abscisic acid (ABA), 100 µM salicylic acid (SA), 30 µM methyl jasmonate (MJ), 1 µM cytokinin (CK) or 10 µM indole-3-acetic acid (IAA; auxin) (Kakei *et al*., 2015). DEG = differentially expressed genes. 'DEG with rice orthologs' lists the Brachypodium genes that had a clear ortholog in rice.

Hormome	DEG in Brachypodium	DEG with rice orthologs	Found in *Lr34res* core gene set	Expected*	p-value
**ABA**	**445**	**409**	**25**	**3.35**	**<0.0001**
MJ	383	352	5	2.89	0.21
IAA	125	118	0	1	0.32
SA	81	75	0	0.5	0.48
CK	23	22	0	0	-

* Number of genes expected to be found in the '*Lr34res*-responsive core gene set' if there were no enrichment for a particular hormone. The values were calculated based on the number of 'DEG with rice orthologs' and the total number of genes (19,399) identified in the hormone study (Kakei *et al*., 2015). For example, 409 DEG for ABA represent 2.1% of the total genes. 2.1% of 159 *Lr34res*-responsive genes correspond to 3.35 genes that would be expected in the '*Lr34res*-responsive core gene set' by chance. The p-value was calculated based on the expected and observed genes using a Chi-square test.

Table S6. P-values of the generalized linear analysis based on the leaf sheath assay shown in Fig. 5c. P-values < 0.05 are marked in grey.

Level-wise generalized linear model

Comparison	p-value Level 1	p-value Level 2	p-value Level 3	p-value Level 4
19sib x 27-3 - 19 x wt	0.0005	0.9860	0.0015	0.0290
19 x 27-3 - 19 x wt	< 0.001	< 0.001	< 0.001	0.42957
19sib x wt - 19 x wt	< 0.001	< 0.001	< 0.001	< 0.001
19 x 27-3 - 19sib x 27-3	< 0.001	< 0.001	< 0.001	0.00294
19sib x wt - 19sib x 27-3	< 0.001	< 0.001	< 0.001	0.23143
19sib x wt - 19 x 27-3	< 0.001	0.6780	< 0.001	< 0.001

References

Claes B, Dekeyser R, Villarroel R, Vandenbulcke M, Bauw G, Vanmontagu M, Caplan A. 1990. Characterization of a rice gene showing organ-specific expression in response to salt stress and drought. *Plant Cell* **2**(1): 19-27.

Duan JZ, Zhang MH, Zhang HL, Xiong HY, Liu PL, Ali J, Li JJ, Li Z. 2012. *OsMIOX*, a myo-inositol oxygenase gene, improves drought tolerance through scavenging of reactive oxygen species in rice (*Oryza sativa* L.). *Plant Science* **196**: 143-151.

Frandsen G, MullerUri F, Nielsen M, Mundy J, Skriver K. 1996. Novel plant Ca^{2+}-binding protein expressed in response to abscisic acid and osmotic stress. *Journal of Biological Chemistry* **271**(1): 343-348.

Garg R, Tyagi AK, Jain M. 2012. Microarray analysis reveals overlapping and specific transcriptional responses to different plant hormones in rice. *Plant Signaling & Behavior* **7**(8): 951-956.

Huang X, Wang J, Du Z, Zhang C, Li L, Xu ZQ. 2013. Enhanced resistance to stripe rust disease in transgenic wheat expressing the rice chitinase gene *RC24*. *Transgenic Research* **22**(5): 939-947.

Kakei Y, Mochida K, Sakurai T, Yoshida T, Shinozaki K, Shimada Y. 2015. Transcriptome analysis of hormone-induced gene expression in *Brachypodium distachyon*. *Scientific Reports* **5**: 14476.

Li L, Li N, Song SF, Li YX, Xia XJ, Fu XQ, Chen GH, Deng HF. 2014. Cloning and characterization of the drought-resistance *OsRCI2-5* gene in rice (*Oryza sativa* L.). *Genetics and Molecular Research* **13**(2): 4022-4035.

Maruyama K, Urano K, Yoshiwara K, Morishita Y, Sakurai N, Suzuki H, Kojima M, Sakakibara H, Shibata D, Saito K, et al. 2014. Integrated analysis of the effects of cold and dehydration on rice metabolites, phytohormones, and gene transcripts. *Plant Physiology* **164**(4): 1759-1771.

Pang ZQ, Zhou ZZ, Yin DD, Lv QM, Wang LX, Xu X, Wang J, Li XB, Zhao XF, Jiang GH, et al. 2013. Transgenic rice plants overexpressing BBTI4 confer partial but broad-spectrum bacterial blight resistance. *Journal of Plant Biology* **56**(6): 383-390.

Perez-Diaz J, Wu TM, Perez-Diaz R, Ruiz-Lara S, Hong CY, Casaretto JA. 2014. Organ- and stress-specific expression of the *ASR* genes in rice. *Plant Cell Reports* **33**(1): 61-73.

Risk JM, Selter LL, Chauhan H, Krattinger SG, Kumlehn J, Hensel G, Viccars LA, Richardson TM, Buesing G, Troller A, et al. 2013. The wheat *Lr34* gene provides resistance against multiple fungal pathogens in barley. *Plant Biotechnology Journal* **11**(7): 847-854.

Shaik R, Ramakrishna W. 2014. Machine learning approaches distinguish multiple stress conditions using stress-responsive genes and identify candidate genes for broad resistance in rice. *Plant Physiology* **164**(1): 481-495.

Wang MB, Boulter D, Gatehouse JA. 1992. A complete sequence of the rice sucrose synthase-1 (*RSs1*) gene. *Plant Molecular Biology* **19**(5): 881-885.

Xiao BZ, Huang YM, Tang N, Xiong LZ. 2007. Over-expression of a *LEA* gene in rice improves drought resistance under the field conditions. *Theoretical and Applied Genetics* **115**(1): 35-46.

Zheng J, Wang YY, He YA, Zhou JJ, Li YP, Liu QQ, Xie XZ. 2014. Overexpression of an S-like ribonuclease gene, *OsRNS4*, confers enhanced tolerance to high salinity and hyposensitivity to phytochrome-mediated light signals in rice. *Plant Science* **214**: 99-105.
